# Evaluation of *LOXL1* polymorphisms in primary open-angle glaucoma in southern and northern Chinese

**Published:** 2008-12-19

**Authors:** Wei Fen Gong, Sylvia W.Y. Chiang, Li Jia Chen, Pancy O.S. Tam, Li Yun Jia, Dexter Y.L. Leung, Yi Qun Geng, Clement C.Y. Tham, Dennis S.C. Lam, Robert Ritch, Ningli Wang, Chi Pui Pang

**Affiliations:** 1 Department of Ophthalmology and Visual Sciences, the Chinese University of Hong Kong, Hong Kong, China; 2 Joint Shantou International Eye Center, Shantou University Medical College, Shantou, China; 3 Department of Ophthalmology, Beijing Tongren Hospital, Beijing, China; 4 Einhorn Clinical Research Center, New York Eye and Ear Infirmary, New York, NY

## Abstract

**Purpose:**

The lysyl oxidase-like protein 1 (*LOXL1*) gene is strongly associated with exfoliation glaucoma, which is very rare in the Chinese population. The implicated *LOXL1* polymorphisms have not been associated with primary open-angle glaucoma (POAG). In this study, we investigated three of the *LOXL1* polymorphisms in POAG in a southern Chinese population of Hong Kong and northern Chinese from Beijing.

**Methods:**

The Hong Kong group included 293 POAG patients and 250 controls, and the Beijing group included 169 POAG patients and 197 controls. *LOXL1* single nucleotide polymorphisms (SNPs), rs1048661, rs3825942, and rs2165241, were genotyped by direct DNA sequencing. Individual association was analyzed using the χ^2^ test, and haplotype-based association analysis was performed in WHAP.

**Results:**

Each of the candidate SNPs was not statistically associated with POAG in either group (p>0.017, Bonferroni correction). Haplotype-based association analysis had identified a significant omnibus association (Omnibus χ^2^=18.16, p=0.00115) between these SNPs and POAG in the Hong Kong group. A minor haplotype (T-G-T) showed significant statistical association with POAG. It presented in 2.1% of cases and 0.4% of controls, conferring a 5.24 fold of increased risk to the disease (95% CI: 1.17–23.54, P_perm_=0.00108). However, this haplotype was absent in the Beijing group.

**Conclusions:**

Individual *LOXL1* SNPs, rs1048661, rs3825942, and rs2165241, were not associated with POAG in the Chinese population. However, a minor haplotype T-G-T was found to be associated with the disorder in the southern Chinese. The low frequencies of the at-risk alleles at rs1048661 and rs2165241 may be one of the factors that led to the low prevalence of exfoliation syndrome in the general populations of the Chinese.

## Introduction

Glaucoma is a group of heterogeneous disorders that can lead to progressive optic neuropathy and loss of vision with or without the association of elevated intraocular pressure (IOP). It is a leading cause of irreversible blindness worldwide [1]. Primary open-angle glaucoma (POAG) is a major form of glaucoma. POAG is a complex disease with multiple genetic risk factors. So far, there are at least 24 candidate loci that have been linked to POAG [2-16]. Three genes have been identified for POAG from the reported loci, myocilin (*MYOC*, OMIM 601652) [17,18], optineurin (*OPTN*, OMIM 602432) [6,19], and WD repeat-domain 36 (*WDR36*, OMIM 609669) [12,20]. However, these genes altogether account for less than 10% of POAG cases [12,18,19].

Recently, in the populations of Iceland and Sweden, three single nucleotide polymorphisms (SNPs; rs2165241, rs3825942, and rs1048661) in the lysyl oxidase-like protein 1 gene (*LOXL1*, OMIM 153456) have been found to be strongly associated with exfoliation glaucoma (XFG, OMIM 177650), which is the most common form of secondary open-angle glaucoma associated with exfoliation syndrome (XFS; OMIM 177650) [21]. Subsequent studies have replicated the association of *LOXL1* SNPs with XFG and XFS among populations in different regions including the United States [22-24], Central Europe [25,26], India [27], and Japan [28-31].

In contrast, rs2165241 in *LOXL1* was only marginally (p=0.04) associated with POAG in the Icelandic population while the other two SNPs, rs3825942 and rs1048661, did not show any significant association [21]. Follow-up studies also demonstrated the lack of association between the *LOXL1* SNPs and POAG in Swedish [21], Australian Caucasian [32], American Caucasian [23], African American [33], Indian [34], and Japanese populations [30,35]. These findings altogether suggested that the *LOXL1* polymorphisms are risk factors for XFS/XFG but not for POAG. However, the role of these *LOXL1* polymorphisms on POAG in Chinese populations is not known, and it is important to look for these SNPs in the general populations of China. The occurrence of XFS/XFG is very infrequent among the Chinese populations compared with the Japanese and Caucasian populations [36,37]. Investigation of the major *LOXL1* SNPs in the general Chinese populations may provide further insight into the discrepancy in the disease prevalence of XFG/XFS between the Chinese and other ethnic groups.

In the Chinese population, we had previously mapped a POAG locus to 15q22-q24 (GLC1N) within a genetic distance of 16.6 Mb flanked by D15S1036 and rs922693 [14]. *LOXL1* is located in this genetic region. Therefore, it is desirable to evaluate the possibility of *LOXL1* being a POAG candidate gene in the Chinese. We have previously found different *MYOC*, *OPTN*, and *WDR36* mutation patterns between Chinese and Caucasian POAG patients [38-40]. Whether the lack of association between *LOXL1* and POAG in other populations also occurs in the Chinese populations requires further investigation. Furthermore, we have recently found that the distributions of variants in *MYOC*, *OPTN*, and *WDR36* are different between Hong Kong Chinese (southern Chinese) and a northern Chinese population from Beijing (unpublished data). In this present study, we investigated the association between the three *LOXL1* SNPs (rs2165241, rs3825942, and rs1048661) and POAG in two groups of Han Chinese, one from southern China and one from northern China, to explore their distributions.

## Methods

### Study subjects

Unrelated POAG patients and control subjects were recruited from the Eye Clinic of the Prince of Wales Hospital and Hong Kong Eye Hospital in Hong Kong, China. This group represented the southern Chinese group that had been previously studied [40]. Another group of patients and controls were recruited from the Eye Center of Tongren Hospital in Beijing, China. These subjects were from Beijing or regions around Beijing and represented the northern Chinese group. All study subjects were Han Chinese.

In both groups, the same diagnostic criteria for POAG were applied including exclusion of congenital glaucoma, exfoliation syndrome, or other secondary causes (e.g., trauma, uveitis, or steroid-induced glaucoma); gonioscopically open anterior chamber angle (Shaffer grade III or IV); characteristic optic disc changes (e.g., vertical cup-to-disc ratio greater than 0.5, disc hemorrhage, or thin or notched neuroretinal rim); characteristic visual field changes according to Anderson’s criteria [41]; and intraocular pressure (IOP) greater than 22 mmHg in both eyes without medications. IOP was measured by applanation tonometry and visual fields by the Humphrey Field Analyzer (Carl Zeiss Meditec, Dublin, CA) with the Glaucoma Hemifield test. Unrelated control subjects were recruited from participants who attended the eye clinic for conditions such as senile cataract, floaters, and mild refractive errors. They underwent complete ophthalmic examinations and were free from glaucoma and other major eye diseases. IOP was measured and recorded at the time they were recruited, and the past IOP data were collected from their medical records. Only subjects aged 60 or above were recruited. They had a historical IOP less than 22 mmHg and had no first-degree relative with glaucoma. The Hong Kong group included 293 POAG cases and 250 controls. The Beijing group contained 169 POAG cases and 197 controls (Table 1).

**Table 1:** Demographic features of the study populations.

	**Hong Kong cohort (southern Chinese)**		**Beijing cohort (northern Chinese)**	
	**Cases (n=293)**	**Controls (n=250)**	**Cases (n=169)**	**Controls (n=197)**
Female (%)	117 (39.9)	127 (50.8)	37 (21.9)	100 (50.8)
Age range	15–88	60–99	8–82	60–89
Mean age (SD) years	66.8 (12.9)	74.1 (6.8)	39.1 (16.5)	69.4 (6.0)

The study protocol was approved by the ethics committees for human research at the Chinese University of Hong Kong and the Capital Medical University in Beijing. All procedures in this study adhered to the tenets of the Declaration of Helsinki. Informed consent was obtained from all subjects after explanation of the nature of the study. Peripheral venous blood was obtained from the subjects.

### Analysis of *LOXL1* polymorphisms

Genomic DNA was extracted from 200 μl of whole blood using a QIAamp Blood Kit (Qiagen, Hilden, Germany) according to the manufacturer’s instructions. Genotypes of the *LOXL1* SNPs (rs2165241, rs1048661, and rs3825942) were determined by polymerase chain reaction (PCR) followed by direct DNA sequencing using a BigDye Terminator Cycle Sequencing Reaction Kit (version 3.1; Applied Biosystems, Foster City, CA) on an ABI 3130XL DNA sequencer (Applied Biosystems) according to the manufacturer’s protocol. The primers used for amplification and the thermal cycling profiles were shown in Table 2.

**Table 2:** Primer sequences and PCR conditions for *LOXL1* polymorphisms.

**Amplicon**	**Primer sequences**	**MgCl_2_ (mM)**	**Annealing temperature (°C)**	**Size (bp)**
rs3825942 and rs1048661*	F:CCAGGTGCCCGACAACTGG	1.5	60	239
	R:AACCCTGGTCGTAGGTCCGC			
rs2165241	F:TCTAGGGCCCCTTGGAGAATAGG	1.5	60	341
	R:AACTGTGGGGCTCAGGGTAGTGG			

The asterisk indicates that SNPs, rs1048661 and rs3825942, were located in the same amplicon.

### Statistical analysis

SNP genotypes in the case and control samples of the two groups were examined for Hardy–Weinberg equilibrium (HWE) using the χ^2^ test. Allelic or genotypic frequencies between cases and controls were compared by χ^2^ test or Fisher’s exact test. The Bonferroni correction was applied to adjust the significance level in multiple comparisons. A p value of less than 0.017 (equal to 0.05/3) was considered as statistically significant. Odds ratios (OR) were estimated using χ^2^ analysis. SPSS version 15.0 software was used (SPSS Inc., Chicago, IL). Pairwise linkage disequilibrium (LD, D’) estimation was performed using Haploview 4.0 (Daly Lab, Broad Institute, Cambridge, MA). Haplotype-based association analysis was performed using the software, WHAP (version 2.09) [42] according to the author’s instructions. Briefly, after detection of a significant main effect using the omnibus H-1 degree-of-freedom (df) test, conditional analyses were performed to evaluate the effect of individual haplotype. A haplotype-specific (HS) test was performed to detect whether a haplotype has an independent effect after controlling for everything else (a significant HS association was defined as p_HS_<0.05), and a sole-variant test was performed to control for the haplotype and test whether it can explain the total association. A haplotype yielding a p value greater than 0.05 from the sole-variant test indicated that it can explain the total association. To investigate all the haplotypes with a frequency greater than 1%, the frequency threshold was set to 1% with the “–at 1” flag. p values from the omnibus test and haplotype-specific association test were corrected by a permutation test with the “–perm 1000” flag. Statistical significance was defined as a corrected p value (P_perm_) of less than 0.05. We also cross checked the haplotype analysis using PLINK.

## Results

### Distribution of the *LOXL1* SNPs in cases and controls of the study groups

Unequivocal genotypic data were obtained from all study subjects. The allelic and genotypic distributions of the candidate SNPs and evaluation of association were shown in Table 3 and Table 4. In both the Hong Kong and Beijing groups, the genotypic distributions of the three candidate SNPs followed Hardy–Weinberg equilibrium in both POAG and controls. However, no statistically significant association was detected between each of the three SNPs and POAG in either population (p>0.017). No significant difference was detected when samples of the two groups were combined for association analysis. When comparing the allelic and genotypic frequencies of each SNP between Southern and Northern Chinese groups, no statistical difference was detected in cases or in controls (p<0.05/6=0.008 was considered as statistically significant using Bonferroni correction). This indicated that the allelic and genotypic distribution patterns of each SNP were similar between the two Chinese groups.

**Table 3:** Distribution of *LOXL1* polymorphisms in the Hong Kong cohort.

**SNP ID**	**Allele Frequency (%)**			**p value**	**Genotype Frequency (%)**			**p value**
	**Allele**	**Cases (n=586)**	**Controls (n=500)**		**Genotype**	**Cases (n=293)**	**Controls (n=250)**	
rs1048661 (R141L)	T	340 (58.0)	264 (52.8)	0.084	TT	109 (37.2)	69 (27.6)	0.047
	G	246 (42.0)	236 (47.2)		GT	122 (41.6)	126 (50.4)	
					GG	62 (21.2)	55 (22.0)	
rs3825942 (G153D)	G	524 (89.4)	438 (87.6)	0.35	GG	237 (80.9)	193 (77.2)	0.54
	A	62 (10.6)	62 (12.4)		GA	50 (17.1)	52 (20.8)	
					AA	6 (2.0)	5 (2.0)	
rs2165241	C	537 (91.6)	449 (89.8)	0.3	CC	246 (84.0)	199 (79.6)	0.14
	T	49 (8.4)	51 (10.2)		CT	45 (15.4)	51 (20.4)	
					TT	2 (0.7)	0 (0.0)	

**Table 4:** Distribution of *LOXL1* polymorphisms in the Beijing cohort.

**SNP ID**	**Allele Frequency (%)**			**p value**	**Genotype Frequency (%)**			**p value**
	**Allele**	**Cases (n=338)**	**Controls (n=394)**		**Genotype**	**Cases (n=169)**	**Controls (n=197)**	
rs1048661 (R141L)	T	176 (52.1)	198 (50.3)	0.62	TT	42 (24.9)	48 (24.4)	0.77
	G	162 (47.9)	196 (49.7)		GT	92 (54.4)	102 (51.8)	
					GG	35 (20.7)	47 (23.9)	
rs3825942 (G153D)	G	304 (89.9)	341 (86.5)	0. 16	GG	136 (80.5)	146 (74.1)	0.35
	A	34 (10.1)	53 (13.5)		GA	32 (18.9)	49 (24.9)	
					AA	1 (0.6)	2 (1.0)	
rs2165241	C	309 (91.4)	361 (91.6)	0.92	CC	141 (83.4)	164 (83.2)	0.55
	T	29 (8.6)	33 (8.4)		CT	27 (16.0)	33 (16.8)	
					TT	1 (0.6)	0 (0.0)	

### Linkage disequilibrium analysis and haplotype-based association analysis

In the Hong Kong group, the exonic SNPs, rs1048661 and rs3825942, were in strong linkage disequilibrium (D’=0.957, 95% confidence bounds [CB]: 0.86–0.99). SNP rs3825942 and the intronic SNP rs2165241 were also in strong LD (D’=1, 95% CB: 0.55–1.0) while SNPs, rs1048661 and rs2165241, were in moderate LD (D’=0.721, 95% CB: 0.55–0.84). By using the haplotype-based omnibus test, which jointly tested all haplotypes, we observed a significant omnibus association of these three SNPs with POAG (Omnibus χ^2^=18.16, df=4, p=0.00115, P_perm_=0.00399; Table 5). PLINK analysis gave consistent results. Therefore, it was appropriate to proceed to conditional testing. A total of five haplotypes with an overall frequency of greater than 1% were detected. By dissecting the effects of each haplotypes, we obtained two haplotype-specific (HS) associations, H4 and H5. A significant HS association indicated that the haplotype was associated with the disease independent of other haplotypes. The relatively uncommon haplotype, T-G-T (H5), was most strongly associated with POAG (P_perm_=0.0011), conferring a 5.24 fold of increased risk to POAG (95% CI: 1.17–23.54). The estimated frequency of this haplotype (H5) was 2.1% (approximately 12 of 596 haplotype counts) in cases and 0.4% (approximately 2 of 500 haplotype counts) in controls. Due to the low frequencies of the haplotype, we performed Fisher’s exact test to confirm the significance and obtained a p value of 0.027. Another HS association was found between the Haplotype H4 (G-G-T) and POAG. It conferred a 1.6 fold of decreased risk to the disease (P_perm_=0.025, OR=0.62, 95%CI: 0.40–0.97). The haplotype H4 presented in 6.3% of cases and 9.8% of controls. However, by using the haplotype-based sole-variant tests (SV_H_), we clarified the pattern of the haplotype. H5 is the sole haplotype that can explain the omnibus association (SV_H_=0.058; Table 5). When only the two exonic SNPs (rs1048661 and rs3825942) were included into haplotype association analysis, no significant omnibus association was detected (Omnibus χ^2^=2.97, df=2, p=0.227).

**Table 5:** Haplotype analysis of *LOXL1* SNPs in POAG in the Hong Kong cohort.

**Haplotype**	rs1048661	rs3825942	rs2165241	**Haplotype frequency**		**HS test (df=1) p value**	**SV_H_ test p value**	**Odds ratio (95% CI)**
				**POAG**	**Controls**			
H1	T	G	C	0.558	0.524	0.278	0.00336	1.15 (0.91–1.46)
H2	G	G	C	0.257	0.25	0.808	0.000419	1.03 (0.78–1.36)
H3	G	A	C	0.101	0.124	0.253	0.000756	0.80 (0.55–1.16)
H4	G	G	T	0.063	0.098	0.0249	0.00347	0.62 (0.40–0.97)
H5	T	G	T	0.021	0.004	0.00108	0.058	5.24 (1.17–23.54)

All haplotypes with frequency greater than 1% were shown in the table. HS test represented haplotype-specific test, and SV_H_ test represents haplotype-based sole-variant test. The p values from the HS test were corrected by a permutation test by 1000 times of iterations. Omnibus χ^2^=18.16 (df=4); p=0.00115, P_perm_=0.00399; n=293 case and n=250 control subjects.

In the Beijing group, the three SNPs were in strong LD. The pairwise D’ values between each pair of SNPs were 1.0. However, compared with that in the Hong Kong group, no significant omnibus association was detected between the three candidate SNPs and POAG (Omnibus χ^2^=2.52, df=3, p=0.471). The haplotype-specific test also showed that none of the haplotypes was significantly associated with POAG. Moreover, in contrast to the Hong Kong group, haplotype T-G-T was not identified in the Beijing subjects (Table 6). When only the two exonic SNPs were taken into account, there was still no significant main effect detected (Omnibus χ^2^=2.14, df=2, p=0.343). When all samples of the two groups were grouped for haplotype-based association analysis, no significant omnibus association was detected for the three SNPs (n_case_=462, n_control_=447, Omnibus χ^2^=6.81, df=3, p=0.08) nor for the two exonic SNPs (Omnibus χ^2^=4.68, df=2, p=0.097).

**Table 6:** Haplotype analysis of *LOXL1* SNPs in POAG in the Beijing cohort.

**Haplotype**	rs1048661	rs3825942	rs2165241	**Haplotype frequency**		**HS test (df=1)**
				**POAG**	**Controls**	**p value**
B1	T	G	C	0.521	0.503	0.612
B2	G	G	C	0.298	0.279	0.578
B3	G	A	C	0.098	0.135	0.117
B4	G	G	T	0.083	0.084	0.982
B5	T	G	T	-	-	-

The B5 haplotype, although not detected in the Beijing cohort, was listed in the table to make a comparison with the Hong Kong cohort in Table 5. The SV_H_ test was not done in the Beijing cohort because of the non-significant omnibus association. Omnibus X^2^=2.525 (df=3); p=0.471. n=169 case and n=197 control subjects.

## Discussion

The present study investigated the involvement of three *LOXL1* polymorphisms in POAG among Han Chinese populations recruited from Hong Kong (southern China) and Beijing (northern China). Univariate analyses for each of the *LOXL1* SNPs (rs1048661, rs3825942, and rs2165241) revealed that none of the three SNPs was significantly associated with POAG individually, regardless of the localization. The non-significant association between individual *LOXL1* SNPs and POAG was consistent with that reported in other populations including Caucasians [21,23,32], Africans [33], Indians [34], and Japanese [35].

In the present study, all the southern Chinese subjects were localized in Hong Kong, and relative genetic homogeneity among the subjects could be assumed. As for the northern Chinese recruited in Tongren Hospital, they came from Beijing or regions around the city, a relatively large and dispersed region north of the Yellow River. To reduce the influence of historical migrations and genetic admixture between northern and southern Chinese, a northern subject was defined and included only if they and their families assured their nativities to be in this region for at least three generations. By using the Hardy–Weinberg equilibrium test, we observed that all three *LOXL1* SNPs followed HWE in each group. Therefore, concerning the *LOXL1* SNPs, there was no significant genetic admixture within each of the two study populations. When comparing the genotypic distributions of these variants between the two groups, no significant difference was detected, indicating that the distributions of the genotypes of individual *LOXL1* SNPs did not vary across the southern and northern Chinese.

However, LD analysis and haplotype-based association analyses revealed that the combinatorial effect of the *LOXL1* SNPs on POAG was different between the southern and northern Chinese populations. In the southern group, pairwise LD analysis showed that the SNP rs1048661 was in strong LD with rs3825942 (D’=0.957, 95% confidence bounds: 0.86–0.99) but in weaker LD with the intronic SNP rs2165241 (D’=0.721, 95% CB: 0.55–0.84). This provided strong evidence for historical recombination between the exonic SNP rs1048661 and intronic SNP rs2165241 as the upper confidence bound on D’ is less than 0.9 according to the criteria of Gabriel et al. [43]. In the Beijing group, all three SNPs were in strong LD, indicating less chance of recombination among the variants.

Using haplotype-based association analysis, we detected significant omnibus association between the three *LOXL1* SNPs and POAG in the Hong Kong group. Subsequently, the haplotype-specific test and sole-variant test revealed that haplotype T-G-T (H5) was the sole haplotype, which can explain the omnibus association (P_HS_<0.005, P_SV_>0.05; Table 5). Other haplotypes were not associated with POAG. Carriers of haplotype H5 have a 5.24 fold increased susceptibility to POAG compared to non-carriers (95% CI: 1.17–23.54). However, such a high odds ratio might be due to the low frequencies of the haplotype so that the effect of H5 might have been overestimated. Due to the low frequencies of the H5 haplotype with 2.1% in cases and 0.4% in controls, Fisher’s exact test was used to confirm the significance. The results (Fisher’s exact test: p=0.027) suggested that the significant association between the T-G-T haplotype and POAG was less likely found purely by chance. Therefore, the H5 haplotype may be a genetic risk factor of POAG for a small fraction of southern Chinese. In contrast, in the Beijing group, no significant omnibus haplotype-based association was detected, and the T-G-T haplotype was not identified. The association between H5 and POAG could not be replicated in the northern Chinese. Absence of the T-G-T haplotype might be due to the strong LD between the three SNPs so that less haplotypes had been formed. Therefore, the association between the T-G-T haplotype and POAG may occur only in the southern Chinese. In recent literature, this association had not been identified in other populations. Therefore, a larger sample size of southern Chinese is required to replicate the finding and to better evaluate the effect of *LOXL1* H5 haplotype on POAG genetics. A mutational screening of *LOXL1* in the southern Chinese pedigree in which we had mapped the novel glaucoma locus on 15q22–24 [14] should help to evaluate its contributions to the development of glaucoma. POAG is a multigenic disease. The hitherto identified causative or modifier genes, *MYOC*, *OPTN*, and *WDR36*, account for less than 10% of POAG cases [12,18,19], suggesting the existence of other genetic risk factors underlying this disease. Therefore, if this association between the *LOXL1* H5 haplotype is proven to be true, *LOXL1* may be involved in the genetic architecture of POAG in a small fraction of southern Chinese, implying that a subgroup of POAG may share a common etiological pathway to exfoliation glaucoma. So far, the etiology of XFS/XFG is not fully known. Identification of *LOXL1* may provide a breakthrough, but the functional roles of the *LOXL1* variants in XFS/XFG are unclear. Therefore, to what extent POAG and XFG share a common etiology and the effect of the *LOXL1* H5 haplotype on POAG still need to be investigated. It is possible that some patients who have XFS in a subclinical state manifest open-angle glaucoma as the initial sign. In these patients, the fibrillar extracellular materials may be too fine to be detected clinically, but they may accumulate in the trabecular meshwork, thus obstructing the outflow of aqueous humor and leading to increased IOP and subsequently glaucoma. Therefore, these patients might have been misdiagnosed as POAG rather than XFG.

The prevalence of XFS is very low in Chinese populations with 0.4% in Hong Kong Chinese [36] and 0.7% in Singaporean Chinese [37] aged 60 or above. In contrast, the prevalence rate of XFS varied between 0.8% and 3.2% in the Japanese [44,45], about 3.4% in the Australian population [32], and as high as 29% in the Icelandic population [21]. Genetic factors underlying such discrepancy in disease occurrence are unclear. *LOXL1* is so far the most strongly associated gene with XFS/XFG having been reported in various populations [21-32]. We summarized the allele frequencies of the *LOXL1* SNPs in normal subjects and POAG cases among different populations (Table 7) and observed variation in allelic frequencies of these SNPs among different ethnic groups. In the control subjects, the allele frequencies for rs3825942 G were similar among Chinese, Japanese, Icelandic, and Swedish populations (frequency: 0.847–0.879) [21,30] but were slightly lower in the American Caucasians (0.795) [23] and Indian (0.750) populations [34]. The lowest frequency was in the African American population (0.599) [33]. Concerning the relatively low frequency of rs3825942 G in the American Caucasians (0.795) reported by Fan et al. [23], it should be noted that the subjects in their study were with broad ethnic diversity, therefore the allele frequency might be biased. In another study by Challa et al, [46] the allele frequency of rs3825942 G in American Caucasians was 0.844, which is similar to other Caucasian populations. In contrast, allele frequencies of rs1048661 G and rs2165241 T were obviously lower in the Chinese and Japanese populations than other populations. Compared with the Icelandic population [21], the allele frequency of rs1048661 G was about 1.3 fold lower, and the frequency of rs2165241 T was fivefold to eightfold lower in the Japanese [30] and Chinese populations. Therefore, the low frequencies of the two at-risk alleles in the Japanese and Chinese may be a factor that leads to a lower prevalence of XFS in these two populations. However, when comparing the disease prevalence, it is 40−70 fold lower in the Chinese [36,37], and 9−36 fold lower in the Japanese [44,45] than in the Icelandic population [21]. Such distinction in disease prevalence cannot be fully explained by the discrepancies in the at-risk allele frequencies. Therefore, there may be lower penetrance of the *LOXL1* variants in the Japanese and Chinese, leading to lower disease occurrence than other populations. Low penetrance of the *LOXL1* variants was also found in the Australian population [32]. Moreover, the prevalence of XFS is lower in the Chinese than Japanese, although the allele frequencies were similar among the two populations. This indicates that other unidentified environmental or genetic factors may be involved in the etiology of XFS among different populations.

**Table 7:** Summary of the reported allele frequencies of the *LOXL1* polymorphisms in POAG.

**Population**	**Phenotype**	**Allele Frequency**			**Sample Size**	**Reference**
		rs1048661 ** G**	rs3825942 ** G**	rs2165241 ** T**		
Iceland	Control	0.651	0.847	0.473	14474	[21]
	POAG	0.711	0.872	0.55	90	
Sweden	Control	0.682	0.879	0.535	198	[21]
	POAG	0.638	0.863	0.488	200	
U.S Caucasian	Control	0.719	0.795	0.456	88	[23]
	POAG	0.724	0.771	0.412	331	
African American	Control	NA	0.599	0.204	97	[33]
	POAG	NA	0.617	0.237	193	
India	Control	0.695	0.75	0.32	105	[34]
	POAG	0.616	0.83	0.325	112	
Japan	Control	0.493	0.877	0.058	138	[30]
	POAG	0.395	0.911	0.048	60	
Southern China	Control	0.472	0.876	0.102	250	Present study
	POAG	0.42	0.894	0.084	293	
Northern China	Control	0.497	0.865	0.084	197	Present study
	POAG	0.479	0.899	0.086	169	

Allele frequencies of rs1048661 G, rs3825942 G, and rs2165241 T in POAG cases and controls among different populations were displayed in the table. In the referred studies [21,23,30,33,34] and in our present study, the SNPs were not significantly associated with POAG individually. NA: not available.

In summary, each of the three candidate SNPs in *LOXL1* was not associated with POAG in Chinese populations. A minor haplotype (T-G-T) defined by these SNPs was associated with an increased susceptibility of POAG in the southern Chinese population, although further replication study is required. Moreover, in the general populations of the Chinese, the low frequencies of the at-risk alleles at rs1048661 and rs2165241 may be one of the factors that lead to a lower prevalence of XFS/XFG in the Chinese.
